# Clinical Outcomes of an Innovative Poly‐L‐Lactic Acid (LASYNPRO) in Facial Rejuvenation: Prospective, Multicenter Spanish Study

**DOI:** 10.1111/jocd.70753

**Published:** 2026-02-19

**Authors:** Fernando Urdiales‐Gálvez, Paula A. Benítez, Iratxe Díaz

**Affiliations:** ^1^ Instituto Médico Miramar Málaga Spain; ^2^ Clínica ROBEGA Madrid Spain; ^3^ Clínica Iratxe Díaz Bilbao Vizcaya Spain

**Keywords:** aesthetic medicine, bioregeneration, elastography, facial rejuvenation, PLLA‐LASYNPRO, poly‐L‐lactic acid, ultrasonography

## Abstract

**Objective:**

To evaluate the clinical efficacy, patient satisfaction, safety profile, and biological impact of Poly‐L‐lactic acid (PLLA LASYNPRO) over 6 months.

**Methods:**

This prospective, multicenter study included patients treated with PLLA‐LASYNPRO. The primary end‐point was the proportion of participants who achieved a reduction of at least 1 point in the Wrinkle‐Severity‐Rating‐Scale (WSRS). Secondary end‐points included the proportion of participants achieving at least a 1‐point reduction in the Midface‐Volume‐Deficit‐Scale (MFVDS), the degree of patient satisfaction assessed by the Global‐Aesthetic‐Improvement‐Scale (GAIS), treatment‐related biological and structural changes, and the incidence of adverse events (AEs).

**Results:**

Thirty‐six female subjects were enrolled. Right‐side treatment success rates increased from 44.4% at month‐1 to 71.9% at month‐6 (Cochran's *Q*, *p* = 0.005); left‐side rates increased from 44.4% to 68.8% (*p* = 0.006). MFVDS‐treated‐side success increased significantly (44.4% to 65.6%; *p* = 0.004). GAIS ratings of “Much Better” or “Better” were reported by 66.7% at month 1 and remained high (65.6% at month‐6). Ultrasound and elastography imaging confirmed increased tissue density, reduction of the Sub‐Epidermal‐Low‐Echogenic‐Band (SLEB), signs of neocollagenesis, and improved viscoelasticity. Serum P1CP levels rose significantly from 134.6 ± 98.9 ng/mL to a peak of 233.2 ± 163.1 ng/mL at month 2 (*p* < 0.001 vs. baseline), remaining elevated through month 6 (*p* = 0.012). AEs were limited to mild–moderate, self‐resolving inflammation, erythema, edema, and injection‐site discomfort; no serious AEs occurred.

**Conclusion:**

PLLA‐LASYNPRO achieved significant, sustained improvements in facial wrinkles and volume with high patient satisfaction and biological evidence of collagen stimulation. The treatment demonstrates an excellent safety profile. Further large‐scale, randomized trials are recommended to confirm long‐term efficacy and define its role in aesthetic practice.

## 1 Introduction

Poly‐L‐lactic acid (PLLA) has been widely applied in regenerative medicine and tissue engineering due to its biocompatibility, biodegradability, and ability to stimulate extracellular matrix (ECM) regeneration [1, 2, 3, 4, 5]. It serves as a foundational material in scaffolds used for bone and cartilage repair, wound healing, and soft tissue regeneration. Through its degradation into lactic acid—a natural metabolite—PLLA promotes cellular signaling that supports tissue remodeling [1, 2, 3, 4, 5].

Based on these regenerative properties, PLLA has been successfully adapted for aesthetic medicine as a biostimulatory agent capable of inducing neocollagenesis and long‐term tissue remodeling [6, 7, 8]. Initially approved in Europe in 1999 as New‐Fill, and subsequently in the United States in 2004, PLLA was indicated for correcting volume loss associated with aging and HIV‐related lipoatrophy [9, 10, 11, 12]. Over time, its clinical use has expanded to include facial contouring, dermal rejuvenation, and body treatments such as those targeting the hands, neck, abdomen, and gluteal regions [7, 8, 13, 14, 15, 16]. In both injectable and thread‐based applications, PLLA promotes fibroblast activation and collagen production, offering minimally invasive, durable improvements in skin quality and structure [15, 17, 18].

The conventional mechanism underlying PLLA efficacy was traditionally attributed to a controlled foreign‐body response, in which low‐grade chronic inflammation activated fibroblasts and stimulated collagen deposition. Classical formulations typically consisted of porous, irregularly shaped microspheres designed to elicit this immune‐mediated response. However, variability in individual immune reactivity was shown to influence clinical outcomes and was associated with adverse events such as nodules or granulomatous reactions [6, 7, 8, 14, 16, 19, 20, 21].

To address these limitations, newer PLLA formulations were developed with modified physicochemical characteristics, including uniform microsphere geometry and reduced porosity, with the aim of promoting collagen synthesis through lower‐inflammatory, bioinductive mechanisms. Preclinical investigations suggested that such formulations supported tissue regeneration while minimizing excessive immune activation, thereby shifting the regenerative process from inflammation‐driven remodeling toward more controlled, signal‐mediated pathways [17, 22, 23, 24]. One such formulation was incorporated into the CE‐marked medical device JULÄINE (Juläine, Nordberg Medical AB, Sweden) and was based on PLLA‐LASYNPRO technology.

Preliminary findings from this ongoing investigation were previously reported, demonstrating favorable outcomes in terms of aesthetic improvement and safety following treatment with this novel PLLA formulation [25]. The initial data indicated that most adverse events were mild and transient, with a low incidence of moderate events. In addition, high patient satisfaction and significant increases in procollagen type I C‐terminal propeptide (P1CP) levels supported active neocollagenesis and overall aesthetic enhancement [25]. These findings suggested a favorable safety profile and promising regenerative potential.

The current study aimed to evaluate the clinical performance of this new‐generation PLLA formulation in facial rejuvenation, with a particular emphasis on correcting volume deficits and addressing skin laxity in the nasolabial folds. Additionally, the safety profile of the treatment was assessed through systematic evaluation of adverse event incidence.

## 2 Methods

### 2.1 Study Design

This study was a prospective, multicenter, open‐label, non‐randomized, and non‐controlled single‐arm design trial conducted on patients who underwent treatment with the novel PLLA‐LASYNPRO (Juläine, Nordberg Medical AB, Sweden). Data analysis was performed in a blinded manner to maintain the analyst's impartiality regarding the study intervention.

The study was conducted in accordance with Good Clinical Practice standards, the Declaration of Helsinki, and all relevant national regulations, applying the most stringent criteria to safeguard participant welfare. Prior to inclusion, written informed consent was obtained from each participant. To preserve confidentiality, all personal identifiers were anonymized or encrypted as required. The study protocol received approval from the Ethics Committee of the Instituto Médico Miramar.

### 2.2 Study Participants

The study enrolled adult male and female subjects, who were attended in one of the three participant centers, exhibiting facial volume loss and skin laxity localized to the nasolabial folds. Eligible participants were 18 years of age or older, immunocompetent, and capable of providing written informed consent. All subjects had mild to severe nasolabial folds, defined by a Wrinkle Severity Rating Scale (WSRS) [26] score of ≥ 2 on both sides of the face. Participants were also required to comply with all study‐related procedures and to avoid any additional facial treatments during the follow‐up period.

Exclusion criteria included prior surgical procedures or dermal filler injections in the nasolabial region, recent localized therapies to facial areas below the zygomatic arch, pigmentary alterations or hypomelanosis in the nasolabial area, a tendency toward keloid or hypertrophic scarring, and known allergies to study‐related components such as lidocaine or PLLA. Additional exclusions comprised a history of facial herpes outbreaks, malignant skin conditions, systemic illnesses, coagulation disorders, use of anticoagulant medications, recent immunosuppressive or corticosteroid therapy, impaired wound healing capacity, pregnancy or lactation, and any intent to conceive during the study period. Participants with any condition that could compromise adherence to study procedures or data reliability were excluded at the investigator's discretion.

### 2.3 Treatment Protocol

Participants received treatment following the approved product guidelines, which consisted of up to three administrations of PLLA‐LASYNPRO (Juläine, Nordberg Medical AB, Sweden). Injections were performed into the deep dermal or subcutaneous tissue of the nasolabial folds at baseline, week 4, and week 8, each within a ±1‐week time window.

Follow‐up assessments were scheduled 1 week (±1 day) after each injection to monitor safety, and at 1, 3, and 6 months after the final injection to evaluate both safety and efficacy. The maximum participation duration for each subject did not exceed 10 months. Detailed information of the treatment protocol has been published elsewhere [25].

### 2.4 Injection Technique

Subcutaneous injections of PLLA‐LASYNPRO were performed using a 25G blunt‐tip cannula, following a retrograding (fan‐shaped) technique to ensure even product distribution across the treatment area. The lyophilized PLLA was reconstituted with 5 mL of sterile saline solution per vial, according to manufacturer instructions and under aseptic conditions. The vial was swirled or rotated for 1 min to suspend the powder before injection. A total volume of 1.5 cc per side was injected, with approximately 0.1 cc delivered per injection point. Following injection, firm planar pressure was applied to reduce the risk of cord or nodule formation, and the area was massaged in circular motions for approximately 2 min to facilitate uniform dispersion of the product and enhance tissue integration (Figure S1).

### 2.5 Assessment of Wrinkle and Volume Deficiency Severity

#### 2.5.1 Wrinkle Severity Rating Scale (WSRS)

The WSRS is a validated clinical instrument used to quantify the intensity of facial rhytides through a standardized, five‐grade ordinal scoring system [26]. Each score reflects a distinct level of wrinkle prominence, allowing for consistent clinical interpretation across evaluators:

- Grade 0—None: No visible wrinkles are present.
- Grade 1—Mild: Fine lines are discernible; wrinkles are shallow and minimally apparent.
- Grade 2—Moderate: Wrinkles exhibit intermediate depth and are clearly visible.
- Grade 3—Severe: Deep rhytides with well‐demarcated borders are evident.
- Grade 4—Very Severe: Pronounced, elongated wrinkles resulting in marked skin folds.

This scale serves as a reliable metric for monitoring dynamic changes in wrinkle depth over time, particularly in response to therapeutic interventions [26].

#### 2.5.2 Midface Volume Deficit Scale (MFVDS)

The Midface Volume Deficit Scale (MFVDS) is a clinician‐reported, six‐point photonumeric scale developed by Allergan to assess the degree of volumetric depletion in the central facial region [27, 28]. It enables reproducible grading of midface volume loss, contributing to both aesthetic evaluations and clinical decision‐making. The MFVDS includes the following gradations:

- Grade 0—None: Full midface volume with no observable deficit.
- Grade 1—Minimal: Subtle volume reduction without contour irregularities.
- Grade 2—Mild: Mild flattening of the midface contour.
- Grade 3—Moderate: Clear signs of volume loss, with noticeable structural changes.
- Grade 4—Marked: Pronounced midface hollowing with visible sagging.
- Grade 5—Severe: Significant volume depletion with extensive contour collapse.

Detailed descriptors for each classification level have been previously described in detail [25].

### 2.6 Assessment of Patient Satisfaction

Patient‐reported satisfaction with aesthetic outcomes was evaluated at the 6‐month follow‐up using the Global Aesthetic Improvement Scale (GAIS), a validated, five‐point ordinal instrument designed to assess perceived changes in appearance following treatment [25, 29]. Participants were asked to self‐rate their overall facial aesthetic outcome relative to baseline, categorizing their improvement as one of the following:

- “Very much improved” [5]: Optimal cosmetic result with significant improvement.
- “Much improved” (4): Marked enhancement from baseline but not optimal.
- “Improved” (3): Moderate improvement, yet not fully satisfactory.
- “No change” (2): No perceptible difference compared to baseline.
- “Worse” (1): Deterioration in appearance.

### 2.7 Ultrasonographic Assessment

All sonographic evaluations were conducted by a single, highly experienced examiner (FUG), employing the Samsung HT 30 diagnostic ultrasound system (Samsung Healthcare Global, Gangwon, South Korea), equipped with a 12 MHz linear‐array transducer.

Ultrasound imaging was carried out at baseline (pre‐intervention), as well as at 30, 90, and 180 days following the first therapeutic intervention.

Additionally, ultrasonography was utilized to examine morphological alterations in the dermis and the underlying subcutaneous connective tissue.

### 2.8 Elastographic Evaluation

Cutaneous strain elastography of the bilateral preauricular regions was performed using the ElastoScan module (HS30/XH30, Samsung Healthcare Global, Gangwon, South Korea) prior to treatment and at 60‐, 90‐, and 180‐days post first treatment dose to assess viscoelastic properties and tissue stiffness dynamics.

### 2.9 Evaluation of Aesthetic Outcomes

Aesthetic results were assessed utilizing both two‐dimensional (2D) and three‐dimensional (3D) photographic imaging conducted at baseline and at 30 days, 60 days, 90 days, and 180 days post first‐dose intervention.

Dermal tightening effects were quantitatively analyzed through variations in Facial Tension Vectors at 60, 90, and 180 days following the first‐treatment dose.

#### 2.9.1 2D Imaging

Standardized 2D photographs were acquired using the Sony DSC‐HX400V imaging device (Sony Group Corporation, Konan Minato‐ku, Tokyo, 108–0075, Japan).

#### 2.9.2 3D Imaging

Three‐dimensional volumetric assessments were performed using the Vectra H2 photogrammetric scanning system (Canfield Scientific Inc., Parsippany, NJ, USA). The imaging protocol involved the sequential acquisition of multiple photographs at each designated follow‐up interval, in accordance with the manufacturer's operational guidelines. These images were algorithmically integrated to generate 3D reconstructions.

Furthermore, the Vectra H2 system was employed to quantify dermal tightening by analyzing deviations in Facial Tension Vectors relative to baseline measurements.

### 2.10 Study Outcomes

The primary endpoint was the proportion of participants who achieved a reduction of at least 1 point in the WSRS score 6 months following the final injection.

Secondary endpoints included:

- Proportion of subjects with a ≥ 1‐point WSRS reduction at 3‐ and 4‐months post‐treatment.
- Proportion of patients demonstrating a ≥ 1‐point improvement in the MFVDS at month 6.
- Proportion of participants rated as “much better” or “much improved” on the GAIS at month 6.
- Variations in serum levels of procollagen type I carboxy‐terminal propeptide (P1CP) throughout follow‐up.
- Incidence and characterization of adverse events (AEs).
- Assessment of product integration and neocollagenesis using ultrasonography.
- Evaluation of skin elasticity via ultrasound elastography.
- Aesthetic improvements in volume, tightening, and skin texture of the nasolabial fold region, as determined by Vectra H2 3D imaging and 2D photographic assessment.

### 2.11 Statistical Analysis

#### 2.11.1 Sample Size Calculation

Sample size estimation assumed that 75% of participants would achieve at least a 1‐point WSRS improvement at 6 months compared to baseline. With a type I error (α) of 0.05 and a type II error (β) of 0.20, the required sample size was calculated to be 29 subjects. To account for an anticipated dropout rate of approximately 20%, a total of 36 participants were recruited.

#### 2.11.2 Data Analysis

All statistical analyses were conducted using MedCalc Statistical Software version 23.2.6 (MedCalc Software Ltd., Ostend, Belgium; https://www.medcalc.org; 2025). Data were expressed as medians with 95% confidence intervals (CI), interquartile ranges (IQR), means ± standard deviation (SD), or percentages, depending on variable type.

Changes in P1CP levels over time were analyzed using Friedman's two‐way analysis of variance for repeated measures. The Conover post hoc method was applied for pairwise comparisons.

Cochran's *Q* test was employed to evaluate differences in the proportion of patients classified as treatment successes based on the WSRS across multiple time points during the study. Additionally, this statistical method was applied to assess variations over time in the proportion of patients who achieved a ≥ 1‐point improvement on the MFVDS, as well as those rated as “very much improved” or “much improved” on the GAIS throughout the follow‐up period. Pairwise comparisons between variables were performed in accordance with the procedure outlined by Sheskin [30].

Chi‐squared tests were used to assess categorical variables. A *p*‐value of < 0.05 was considered statistically significant.

## 3 Results

A total of 36 individuals (12 patients per study center), all women, were included in the study. Four patients discontinued the study after being lost to follow‐up at the month 4 (3 subjects) and month 6 (1 subject) visits following the first injection. Data from these patients were maintained through month 3, although they were eliminated from the final analyses.

A detailed summary of their efficacy and safety outcomes is provided in Table S1.

Mean age was 50.5 ± 7.7 (95% CI: 47.9 to 53.1) years.

### 3.1 Wrinkle Severity Rating Scale

At baseline, 7 patients (19.5%) had a WSRS score ≤ 3; 28 patients (77.7%) had a score of 3–4, and 1 patient (2.8%) had a score of 5 on both the right and left sides.

**FIGURE 1:**
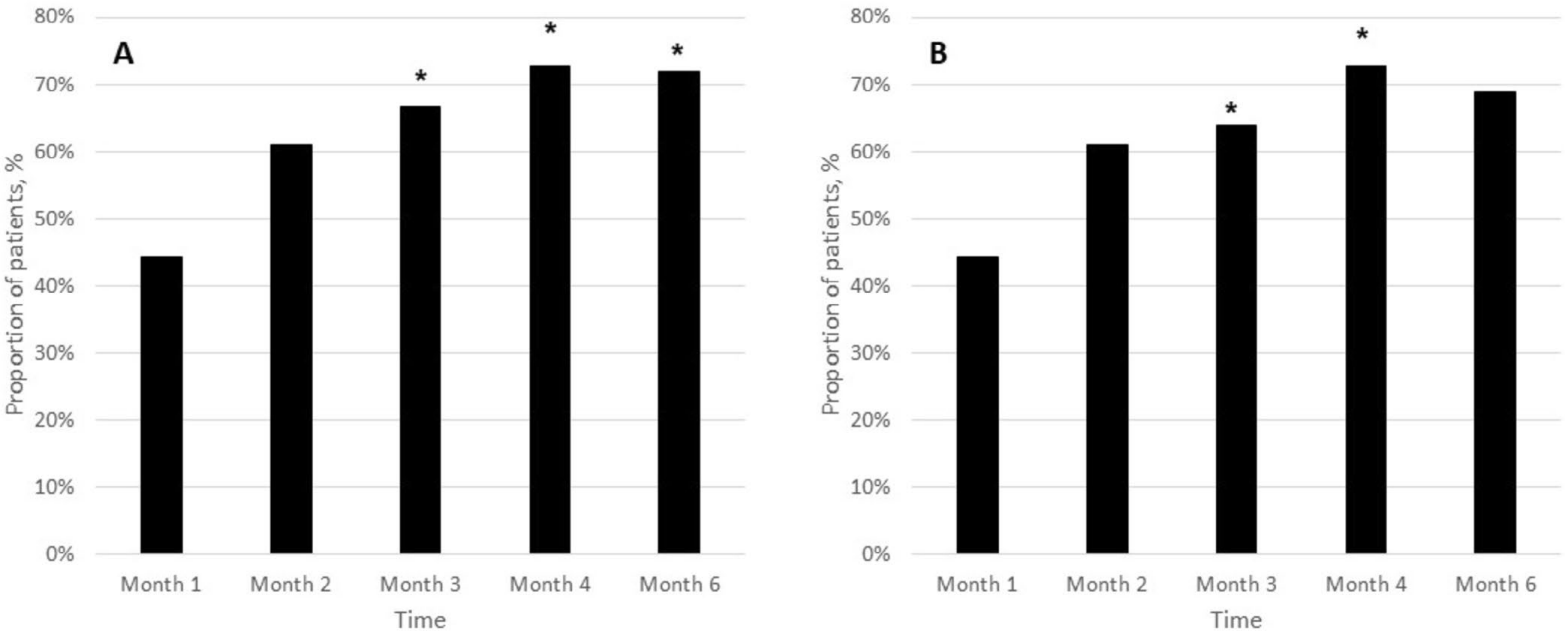
Proportion of patients classified as treatment successes based on the Wrinkle Severity Rating Scale (WSRS) at each follow‐up visit. (A) Right side: Success rates increased from 44.4% (16/36) at month 1 to 61.1% (22/36) at month 2, 66.7% (24/36) at month 3, reaching 75.0% (24/32) at month 4, and to 71.9% (23/32) by month 6. Statistical analysis using Cochran's *Q* test indicated a significant change in treatment success rates over time (*p* = 0.005). (B) Left side: Success rates similarly rose from 44.4% (16/36) at month 1 to 61.1% (22/36) at month 2, 63.9% (23/36) at month 3, peaked at 75.0% (24/32) at month 4, and to 68.9% (22/32) at month 6. This temporal trend was also statistically significant (Cochran's *Q* test, *p* = 0.006). *p < 0.05 versus month‐1.

On the right side, the proportion of treatment successes was 44.4% (16/36) at month 1, increasing to 61.1% (22/36) at month 2, 66.7% (24/36) at month 3, 72.7% (24/33) at month 4, and 71.9% (23/32) at month 6. This temporal improvement was statistically significant (Cochran's *Q* test, *p* = 0.005) (Figure 1A).

On the left side, treatment success rates rose from 44.4% (16/36) at month 1 to 61.1% (22/36) at month 2, 63.9% (23/36) at month 3, 72.7% (24/33) at month 4, and 68.8% (22/32) at month 6 (Cochran's *Q* test, *p* = 0.006) (Figure 1B).

### 3.2 Midface Volume Deficit Scale

At baseline, 7 patients (19.5%) had an MFVDS score ≤ 3; 27 (75.0%) scored 3–4, and 2 (5.6%) scored 5 on both facial sides.

**FIGURE 2:**
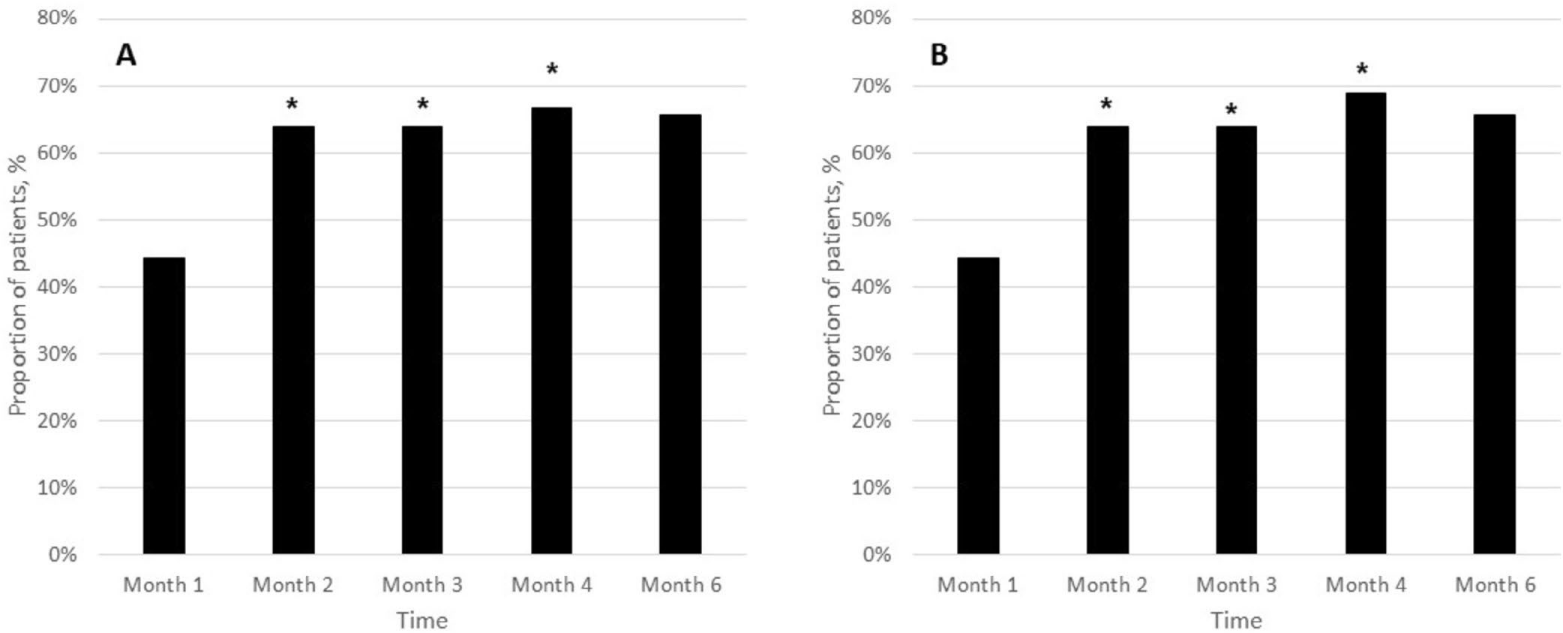
Proportion of patients achieving a ≥ 1‐point reduction in the Midface Volume Deficit Scale (MFVDS) at each follow‐up visit, on both the right (A) and left (B) sides. *t* month 1, 16 patients (44.4%) were considered treatment successes. This proportion increased to 63.9% (23/36) at month 2, remained at 63.9% (24/36) at month 3, rose to 68.8% (22/32) at month 4, and slightly declined to 65.2% (21/32) at month 6. The increase in treatment success over time was statistically significant for both sides (Cochran's *Q* test, *p* = 0.004 for each side, respectively). *p < 0.05 versus month‐1.

Treatment success was achieved in 44.4% (16/36) of patients on both sides at month 1. This proportion increased to 63.9% (23/36) at month 2, remained stable at 63.9% (24/36) at month 3, rose to 66.7% (22/33) at month 4, and slightly declined to 65.6% (22/32) at month 6. The increase over time was statistically significant on both sides (Cochran's *Q* test, *p* = 0.004 for each) (Figure 2A,B).

### 3.3 Assessment of Patient Satisfaction

**FIGURE 3:**
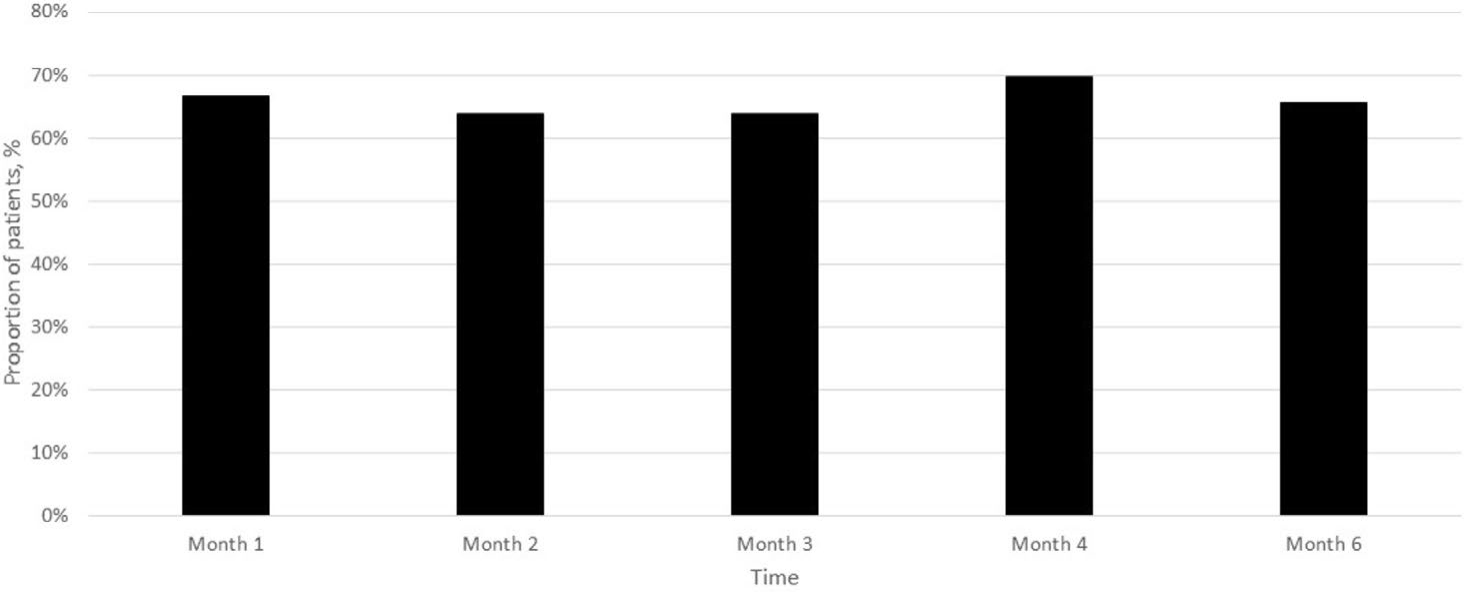
Proportion of patients reporting improvement in aesthetic appearance (“Much Better” or “Better”) on the Global Aesthetic Improvement Scale (GAIS) at monthly intervals following each treatment dose. Evaluations were conducted at 1, 2, 3, 4, and 6 months post‐initial treatment. A consistent trend of positive aesthetic perception was observed across the study period, with the highest satisfaction rate (69.7%) reported at month 4. Percentages are based on the number of patients assessed at each time point (*n* = 36 at months 1–3, *n* = 33 at month 4, and *n* = 32 at month 6).

Patient‐reported satisfaction, assessed using the Global Aesthetic Improvement Scale (GAIS) at each visit, showed a consistently positive trend. At month 1, 66.7% (24/36) of participants rated their appearance as “Much Better” or “Better.” At month 2, 63.9% (23/36) reported further improvement compared to month 1. This proportion remained stable at month 3 (63.9%, 23/36), increased to 69.7% (23/33) at month 4, and was 65.6% (21/32) at month 6 (Figure 3).

**FIGURE 4:**
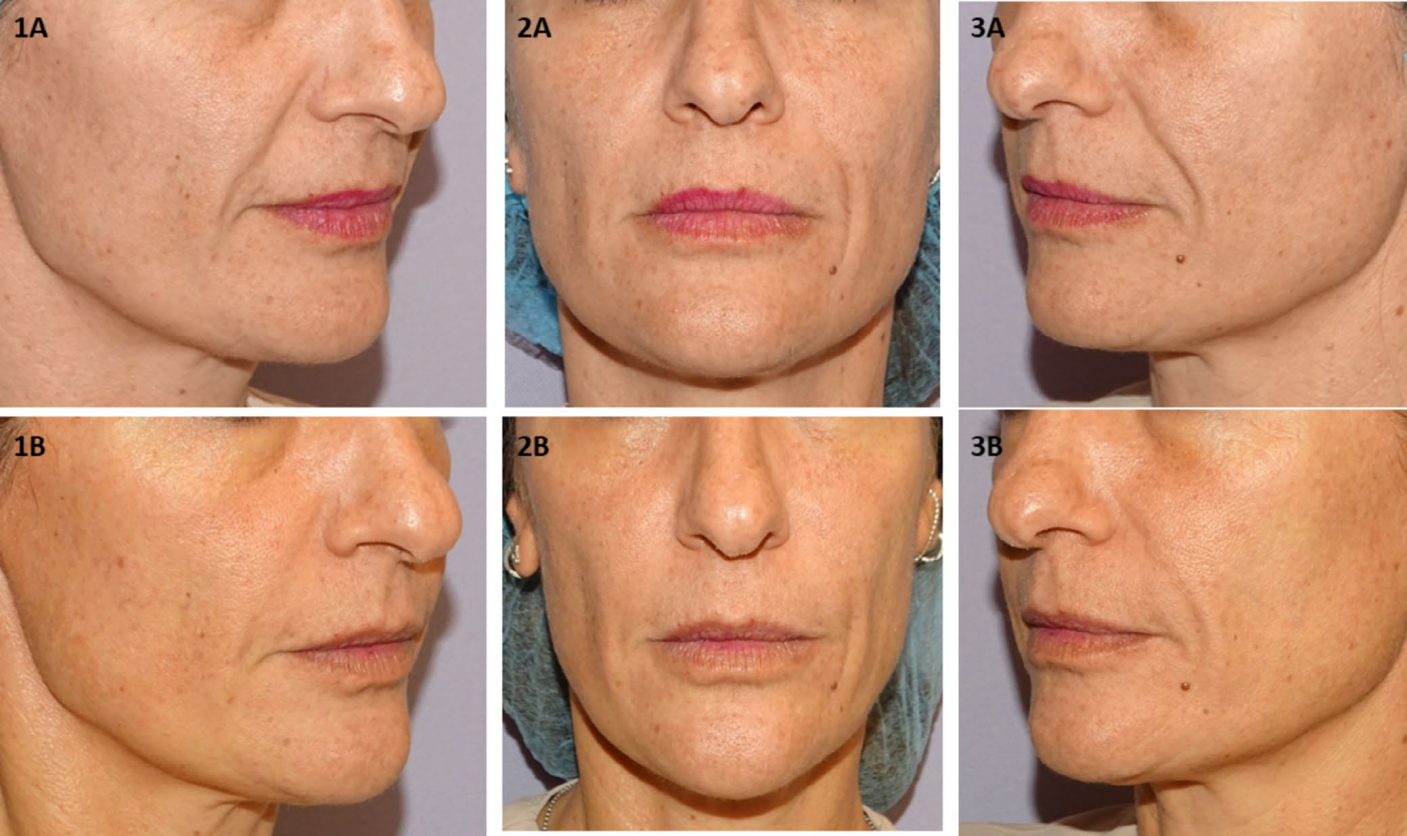
Right (1), frontal (2), and left (3) projections of a 45‐year‐old female patient before (A) and after (B) treatment with PLLA‐LASYNPRO. The patient provided informed consent for the use of her images in this publication. At baseline, the patient (weight: 58 kg) had no prior medical history or previous aesthetic treatments. Clinical evaluation revealed an Ascher grade IV with retrusion grade II, consistent with advanced midface volume loss and moderate skeletal retrusion. Objective assessment using validated clinical scales demonstrated a Wrinkle‐Severity‐Rating‐Scale (WSRS) score of 3 bilaterally and a Midface‐Volume‐Deficit‐Scale (MFVDS) score of 5 on both sides. Following three treatment sessions (1.5 mL per session, using a 5 mL/vial dilution administered to each nasolabial fold), the six‐month post‐treatment evaluation showed substantial improvement. The WSRS score decreased to 1 bilaterally, indicating minimal wrinkle severity, and the MFVDS score improved to 2 on both sides, corresponding to mild midface volume loss and shallow nasolabial folds. Patient‐reported satisfaction, assessed using the Global‐Aesthetic‐Improvement‐Scale (GAIS), was rated as 5 (“Very much improved”).

**FIGURE 5:**
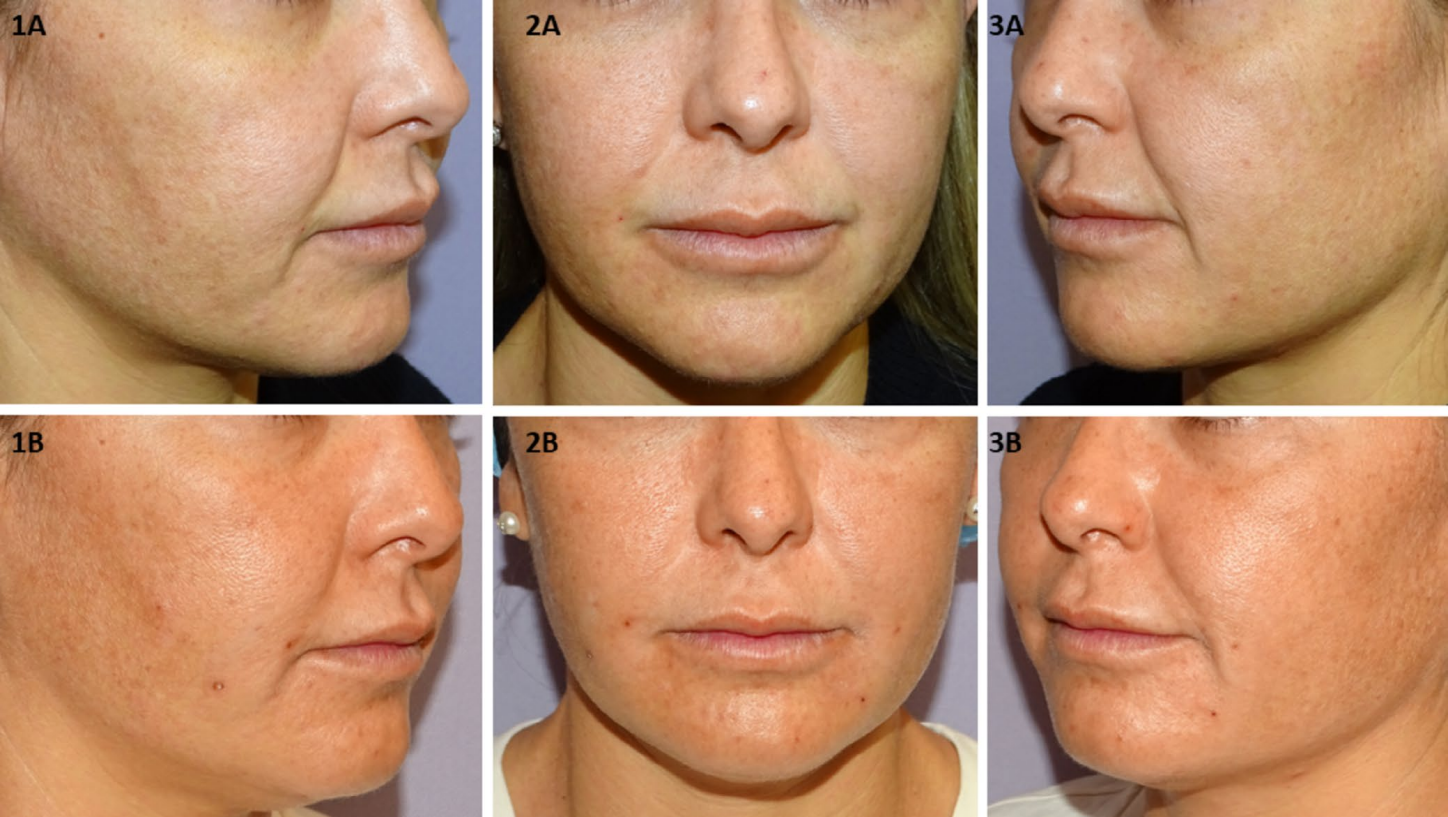
Right (1), frontal (2), and left (3) projections of a 37‐year‐old patient's face before (A) and after (B) treatment. The patient provided consent for the use of their images in this publication. The subject (weight 59 kg) had no prior medical conditions or aesthetic treatments. Pre‐treatment facial assessment revealed an Ascher grade II, indicating mild to moderate midfacial volume loss without significant tissue laxity, and retrusion grade 0, indicating no midface skeletal retrusion. Validated scoring showed a baseline Wrinkle Severity Rating Scale (WSRS) score of 4, corresponding to advanced nasolabial fold severity and a Mid‐Face Volume Deficit Score (MFVDS) of 4 consistent with advance volume loss. Following three treatment sessions (1.5 mL per session, using a 5 mL/vial dilution administered to each nasolabial fold), the six‐month post‐treatment evaluation showed substantial improvement. The WSRS score decreased to 2 bilaterally and the MFVDS score improved to 2 on both sides.

Representative clinical outcomes based on standardized photographs are shown in Figures 4 and 5. Vectra H2 3D imaging analyses further confirmed volumetric improvements relative to baseline (Figures S2–, S4).

**FIGURE 6:**
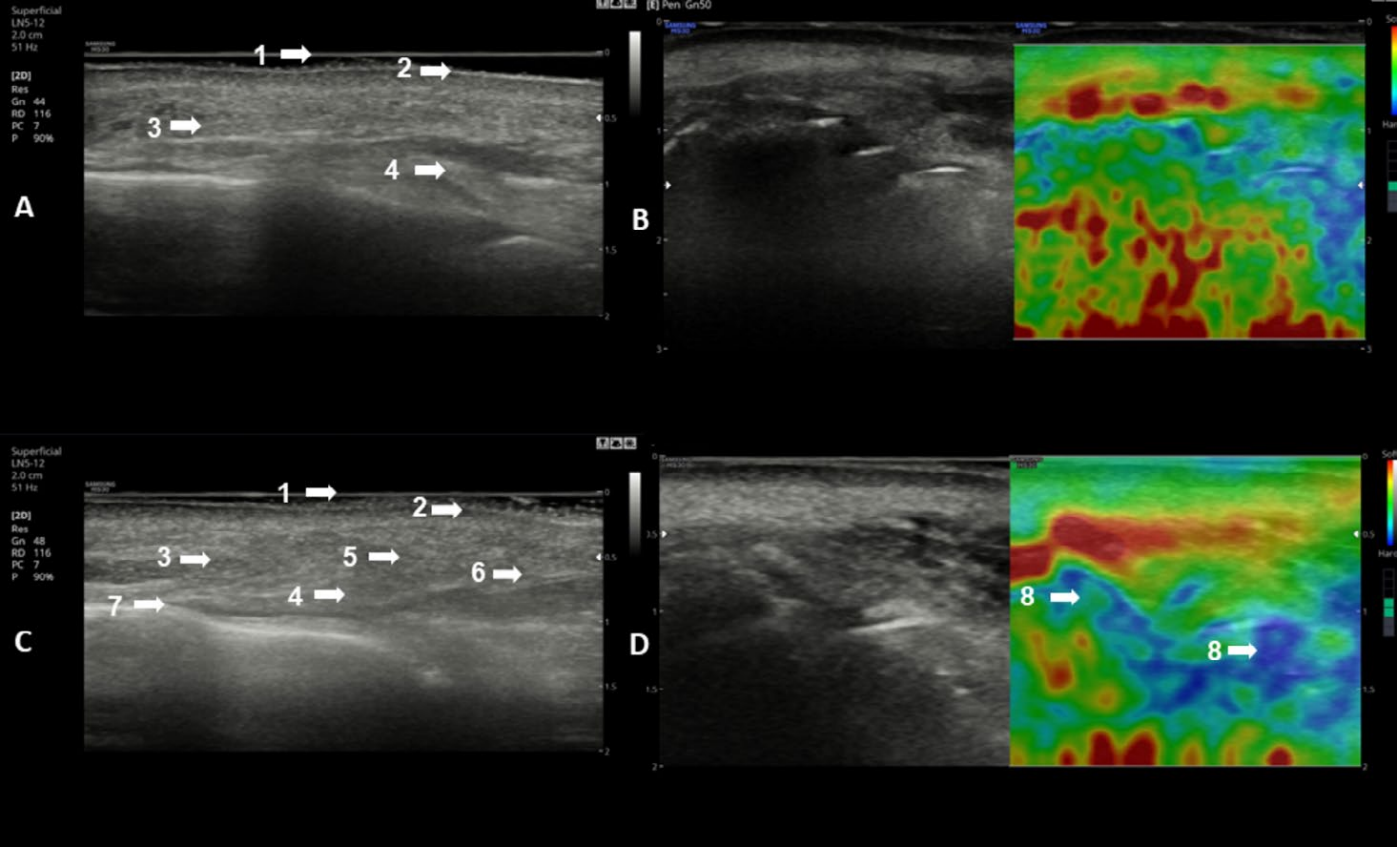
Ultrasound images of the right (R) nasolabial folds at baseline (A) and at 6 months post‐initial treatment (C). Qualitative elastography images of the same region are shown at baseline (B) and at 6 months (D) in the patient presented in Figure 4. At baseline (A), ultrasound revealed a heterogeneous pattern of the nasolabial fold. By month 6 (C), there was a notable increase in tissue density at NLF points 1–2–3, evidenced by the following changes: A reduction in the Sub‐Epidermal Low Echogenic Band (SLEB), accompanied by an increase in echogenicity, indicating higher tissue density (5, 6), despite the persistence of some heterogeneity (7). Evidence of neocollagenesis at the level of the reticular dermis, particularly at its interface with the subcutaneous tissue (5, 6). Areas of increased viscoelasticity, corresponding to a higher elastic modulus, were visualized in blue on qualitative elastography (8).

**FIGURE 7:**
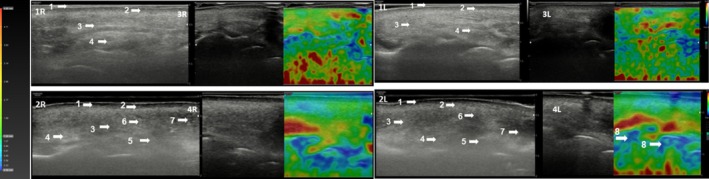
Ultrasound images of the right (R) and left (L) nasolabial folds at baseline (1R & 1L) and at 6 months post‐initial treatment (2R & 2L). Qualitative elastography images of the same region are shown at baseline (3R & 3L) and at 6 months (4R & 4L) in the patient presented in Figure 6. At baseline (1R & 1L), ultrasound revealed a heterogeneous pattern of the nasolabial fold. By month 6 (2R & 2L), there was a notable increase in tissue density at NLF points 1–2–3, evidenced by the following changes: Increased hyperechogenicity is evidenced by the presence of hyperechoic (white) signals within a heterogeneous pattern (5, 6) indicating enhanced tissue density and elevated elastic modulus associated with collagen production (white and light areas). A reduction in the Sub‐Epidermal Low Echogenic Band (SLEB), along with increased echogenicity, further reflects higher tissue density (5, 6), although some residual heterogeneity remains (7). Evidence of neocollagenesis at the level of the reticular dermis, particularly at its interface with the subcutaneous tissue (5, 6). Areas of increased viscoelasticity, corresponding to a higher elastic modulus, were visualized in blue on qualitative elastography (8). 1. Epidermis; 2. Dermis; 3. Subcutaneous Cellular Tissue; 4. Fibrillar Collagen areas; 5. Neocollagen; 6. Hyperechoic Pattern; 7. Anec/Hypoechoic areas; 8. Areas of increased viscoelasticity.

Ultrasound and elastography assessments (Figures 6 and 7) demonstrated structural and biomechanical changes, including increased dermal density, reduction in the sub‐epidermal low echogenic band (SLEB), evidence of neocollagenesis, and enhanced viscoelastic properties in the nasolabial fold region at 6 months compared to baseline.

### 3.4 Safety

During the study, the incidence of AEs was systematically monitored across multiple follow‐up visits (V0–V8). The most frequently reported AE was inflammation, predominantly classified as *mild*, with a peak incidence of 61.1% observed at V1 (1 week post‐first treatment). Mild inflammation persisted variably across subsequent visits but was entirely resolved by V6 (1 month after the third treatment), with no further cases observed through V8 (1 months post‐initial dose). Moderate and severe inflammation were infrequent, each affecting only a small proportion of patients (maximum 6.5% and 2.8%, respectively), and resolved before V6. Erythema occurred sporadically, primarily as mild cases, with incidences ranging from 11.2% to 16.7%, and similarly resolved before V6. Edema was rare and transient, reported in a few cases at V0, V2, and V4, but fully resolved without intervention by V5. Additionally, two subjects reported mild discomfort at the injection site and the presence of a small subcutaneous nodule, both of which resolved spontaneously prior to the subsequent visit. Overall, all adverse events were self‐limited, non‐serious, and resolved without medical treatment or long‐term sequelae.

Table 1 summarizes the incidence and severity of treatment‐related AEs observed throughout the study period.

**TABLE 1:** Incidence of adverse events (AEs).

	V0	V1	V2^a^	V3	V4^b^	V5	V6^c^	V7	V8
Inflammation *n* (%)									
Mild	7 (19.4)	22 (61.1)	18 (33.3)	12 (33.3)	20 (55.6)	14 (38.9)	0 (0.0)	0 (0.0)	0 (0.0)
Moderate	0 (0.0)	0 (0.0)	0 (0.0)	2 (6.5)	0 (0.0)	0 (0.0)	0 (0.0)	0 (0.0)	0 (0.0)
Severe	0 (0.0)	1 (2.8)	0 (0.0)	1 (2.8)	1 (2.8)	0 (0.0)	0 (0.0)	0 (0.0)	0 (0.0)
Edema *n* (%)									
Mild	1 (2.8)	0 (0.0)	0 (0.0)	0 (0.0)	0 (0.0)	0 (0.0)	0 (0.0)	0 (0.0)	0 (0.0)
Moderate	2 (5.6)	0 (0.0)	2 (5.6)	0 (0.0)	2 (5.6)	0 (0.0)	0 (0.0)	0 (0.0)	0 (0.0)
Severe	0 (0.0)	0 (0.0)	0 (0.0)	0 (0.0)	0 (0.0)	0 (0.0)	0 (0.0)	0 (0.0)	0 (0.0)
Erythema *n* (%)									
Mild	5 (13.9)	0 (0.0)	6 (16.7)	0 (0.0)	4 (11.2)	0 (0.0)	0 (0.0)	0 (0.0)	0 (0.0)
Moderate	1 (2.8)	0 (0.0)	1 (2.8)	0 (0.0)	1 (2.8)	0 (0.0)	0 (0.0)	0 (0.0)	0 (0.0)
Severe	0 (0.0)	0 (0.0)	0 (0.0)	0 (0.0)	0 (0.0)	0 (0.0)	0 (0.0)	0 (0.0)	0 (0.0)

*Note:* During the study follow‐up period, two patients reported experiencing “mild discomfort at the injection site” and the presence of “a small bump” in the injection area. Both adverse events were successfully resolved without treatment and sequalae before the next visit. V0: Immediately after the first treatment; V1: One week ±1 day after the first treatment; V2: Before the second treatment; V3: One week ±1 day after the second treatment; V4: Before the third treatment; V5: One week ±1 day after the third treatment; V6: One month after the third treatment (3 months after the first dose); V7: Four months after the first dose; V8: Six months after the first dose.

^a^ One month ±1 week after the first treatment.

^b^ One month ±1 week after the second treatment.

^c^ One month ±1 week after the third treatment.

### 3.5 Procollagen Type I Carboxy‐Terminal Propeptide

At baseline, the mean ± standard deviation (SD) of P1CP was 134.6 ± 98.9 ng/mL. One month after the first dose, P1CP levels increased significantly to 182.4 ± 96.6 ng/mL (mean difference ± standard error [SE]: 47.7 ± 13.7 ng/mL; 95% CI: 19.9 to 75.5 ng/mL; *p* = 0.0014, repeated measures ANOVA). This upward trend continued, with a marked elevation at month 2, where the mean P1CP reached 233.2 ± 163.1 ng/mL (mean difference ± SE: 98.5 ± 27.2 ng/mL; 95% CI: 43.3 to 153.8 ng/mL; *p* = 0.0009). At month 3, P1CP levels remained significantly elevated compared to baseline (mean difference ± SE: 49.8 ± 15.8 ng/mL; 95% CI: 5.7 to 93.9 ng/mL; *p* = 0.0197). Although a decrease was observed between M2 and M3 (mean difference ± SE: −48.8 ± 24.1 ng/mL; 95% CI: −116.0 to 18.6 ng/mL), this change did not reach statistical significance (*p* = 0.3038).

At month 4, P1CP levels remained elevated relative to baseline, but the difference was not statistically significant (mean difference ± SE: 44.0 ± 40.4 ng/mL; 95% CI: −39.5 to 125.6 ng/mL; *p* = 0.2869). Nevertheless, by month 6, a significant increase from baseline was again observed (mean difference ± SE: 70.3 ± 25.7 ng/mL; 95% CI: 17.2 to 123.5 ng/mL; *p* = 0.0117).

Overall, the data suggested a treatment‐related increase in P1CP levels, with a peak response observed at M2. While a partial decline followed, levels remained elevated relative to baseline through M6, suggesting a sustained biological effect.

**FIGURE 8:**
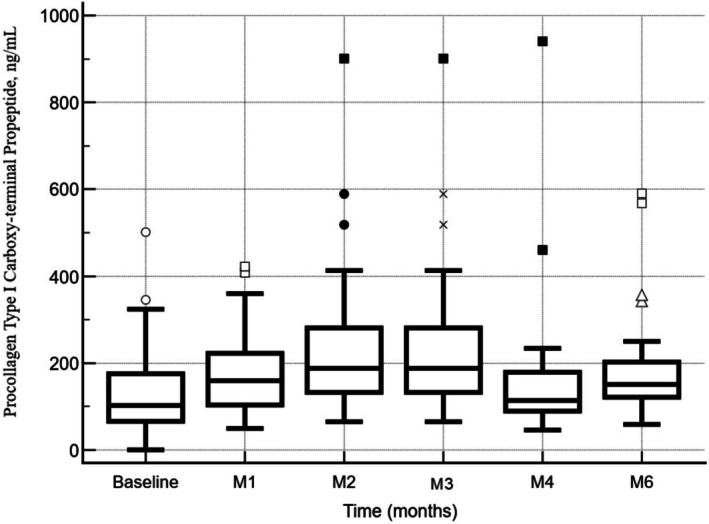
Mean levels of Serum Procollagen Type I Carboxy‐terminal Propeptide (P1CP) throughout the study follow‐up. A significant increase in P1CP was observed at month 1 compared to baseline (mean difference ± standard error [SE]: 47.7 ± 13.7 ng/mL; 95% CI: 19.9 to 75.5; *p* = 0.0014), with a peak response at month 2 (mean difference: 98.5 ± 27.2 ng/mL; 95% CI: 43.3 to 153.8; *p* = 0.0009). Levels remained significantly elevated at month 3 (*p* = 0.0197), although the decrease from month 2 was not statistically significant (*p* = 0.3038). The increase observed at month 4 did not reach statistical significance (*p* = 0.2869), while a significant elevation was again noted at month 6 (mean difference: 70.3 ± 25.7 ng/mL; 95% CI: 17.2 to 123.5; *p* = 0.0117). Data analyzed using repeated measures ANOVA. Baseline, First dose; m, month; M1, Second dose; M2, Third dose.

Figure 8 shows the mean levels of serum P1CP throughout the study follow‐up period.

## 4 Discussion

This prospective, multicenter, open‐label, non‐randomized, and uncontrolled single‐arm study was designed to evaluate the clinical efficacy and safety of PLLA‐LASYNPRO for facial rejuvenation, specifically targeting volumetric enhancement and dermal laxity in the nasolabial region.

Results demonstrated a statistically significant and progressive improvement in wrinkle severity over 6 months, with treatment success rates increasing from 44.4% at 1 month to 71.9% at month six. Secondary outcomes, including midface volume restoration, patient satisfaction, imaging data, and biomarker analysis, were consistent with these findings and suggested a moderate but sustained clinical benefit. Ultrasound and elastography indicated improvements in dermal structure and biomechanical properties, including increased density and collagen deposition.

Unlike traditional dermal fillers that rely on passive volume replacement, PLLA exerts its effect through stimulation of fibroblast activity and neocollagenesis [31]. The transient post‐injection volumizing effect dissipates within days as the carrier fluid is absorbed [32]. Conventional PLLA formulations rely on a controlled foreign body response, inducing low‐grade inflammation that activates fibroblasts and triggers type I collagen production [18, 19, 20, 21, 33, 34]. However, histological studies reveal immune cell infiltration, perivascular fibrosis, and stromal remodeling—hallmarks of reparative rather than regenerative processes [18, 35]. This immune‐mediated mechanism can introduce variability in outcomes and increase the risk of adverse effects such as nodules and granulomas, particularly in susceptible individuals [15, 36]. Contributing to this reactivity are the physicochemical properties of standard PLLA particles, which are irregular in shape and range broadly in size (2–150 μm), making their degradation unpredictable and their immunogenicity higher [37, 38]. Smaller particles (< 20 μm) are readily phagocytosed, further intensifying the inflammatory response [39]. These limitations have necessitated post‐treatment massage protocols, such as the “5 × 5 × 5” rule, to reduce particle aggregation and mitigate adverse reactions [6, 7].

PLLA‐LASYNPRO was engineered to address these limitations by leveraging lactic acid–mediated signaling pathways that modulate fibroblast activity, angiogenesis, and ECM remodeling without eliciting an immune response [17, 22, 23, 24, 40, 41]. The microspheres are uniform, smaller, and non‐porous, making them less prone to phagocytosis and promoting a more predictable degradation and regenerative response [23, 37]. Histological analysis shows simultaneous type I and III collagen synthesis in the absence of foreign body giant cells—indicative of non‐inflammatory remodeling [23]. Transcriptomic profiles support this finding, revealing upregulation of genes associated with ECM renewal and angiogenesis, rather than macrophage‐driven inflammation [42, 43, 44]. Clinically, these properties translate into improved dermal quality, more consistent aesthetic outcomes, diminished inflammation, and the elimination of the need for massage protocols [23, 25, 45].

The present findings are in line with earlier reports [25], reinforcing the clinical efficacy and safety of PLLA‐LASYNPRO. Patient satisfaction remained consistently high throughout the study, and serum P1CP levels—a biomarker of type I collagen synthesis—were significantly elevated at multiple timepoints, peaking at month two and remaining above baseline through month six. These data suggest a sustained stimulatory effect on collagen production.

Complementary imaging at 6 months post‐treatment showed structural improvements, including increased dermal density, reduction in the sub‐epidermal low echogenic band (SLEB), and signs of ongoing neocollagenesis [46]. Enhanced viscoelasticity further confirmed improved skin biomechanics in the treated areas. PLLA has long been recognized as a reliable collagen stimulator with over two decades of clinical use [47]. Fibroblast‐driven collagen synthesis occurs gradually, supporting long‐lasting aesthetic outcomes [48, 49]. Hanako et al. identified type III collagen 16 weeks post‐injection [50], commonly referred to as “baby collagen” or “microcollagen,” which predominates at the dermal‐epidermal junction and contributes to the structural integrity involved in fine wrinkle correction [51, 52].

Our results showing significant improvement in WSRS and MFVDS scores, as well as sustained P1CP elevation over 6 months, align with previous findings on PLLA‐based dermal fillers. Han et al. [53] reported the effectiveness of two different PLLA formulations for correction of the Nasolabial Fold. The results of our study found that PLLA‐LASYNPRO provided comparable or slightly superior improvement in nasolabial folds relative to the new Gana V or Sculptra at 6 months, with a similar safety profile.

The observed improvements in wrinkle severity and volume deficits were also consistent with other studies comparing PLLA or PDLLA to hyaluronic acid. Ting et al. [54] reported that PDLLA achieved greater or equivalent correction of nasolabial folds compared with hyaluronic acid (HA) fillers, with favorable tolerability. Likewise, Hyun et al. [55] found that PLA injections led to noninferior outcomes relative to HA at 6 months, with most adverse events being mild and transient.

When comparing our findings with those reported by Hu and Wang [56] on polycaprolactone (PCL) and PLLA for nasolabial fold correction, our results corroborate the established efficacy of PLLA in achieving progressive wrinkle reduction and volume restoration. Although the assessment methods differed between studies, we observed a substantial and sustained improvement in WSRS and MFVDS scores, consistent with the trends reported by Hu and Wang [56].

Safety outcomes were favorable. Most adverse events were mild, self‐limited, and resolved spontaneously. Inflammation was the most commonly reported event, followed by transient erythema and edema, all resolving without intervention. Moderate or severe events were rare, and no long‐term sequelae were observed.

Although isolated reports cite nodule or granuloma formation following PLLA use, particularly in earlier formulations, these complications have declined significantly with improvements in particle design and injection techniques [13, 23, 57, 58, 59, 60]. Hart et al. reported no nodules in a series of 100 patients receiving thoracic PLLA injections [61]. Common minor side effects such as bruising, swelling, or discomfort typically resolved within 2 weeks post‐treatment [25, 59], and allergic reactions remain rare [62].

This study has limitations. The open‐label, non‐randomized, and uncontrolled design precludes definitive conclusions about causality or comparison with placebo or alternative treatments. Additionally, the sample size was relatively small and limited to female participants, restricting generalizability to broader populations, including males and more diverse demographic groups. Third, the follow‐up period of 6 months may not fully capture long‐term safety and efficacy outcomes. Finally, potential variability in injection technique across study centers might have influenced treatment responses.

Nevertheless, two methodological strengths are notable: (1) the multicenter design enhances external validity by capturing outcomes across varied clinical settings, and (2) the use of blinded data analysis minimizes evaluator bias, thereby reinforcing the credibility of the findings.

## 5 Conclusion

In this prospective multicenter study, PLLA‐LASYNPRO demonstrated a progressive and statistically significant improvement in wrinkle severity and midface volume restoration over a six‐month period, as measured by validated clinical scales. Patient‐reported outcomes aligned with these findings, reflecting high levels of aesthetic satisfaction. Imaging modalities, such as ultrasound, elastography, and 3D photography, corroborated tissue‐level improvements consistent with neocollagenesis and dermal remodeling. Biochemical analysis further supported a sustained increase in type I collagen synthesis. The treatment was well‐tolerated, with only mild to moderate, self‐limiting adverse events and no long‐term complications.

While results are encouraging, further large‐scale, randomized controlled studies are needed to confirm long‐term efficacy and fully establish its role within regenerative aesthetic medicine.

## Author Contributions

F.U.‐G. Designed and directed the project, the main conceptual ideas, and proof outline. P.A.B. and I.D. Drafted the manuscript, literature search, and designed the tables and figures. F.U.‐G., P.A.B., and I.D. collected data. Critical review and edition of the manuscript. All authors reviewed the results and approved the final version of the manuscript.

## Funding

This work was supported by Nordberg Medical Unique Science.

## Ethics Statement

The study protocol received approval from the Ethics Committee of the Instituto Médico Miramar (approval number: IMMPI01‐2024).

## Conflicts of Interest

Dr. Urdiales‐Gálvez received research grants to cover the costs of medical writing services and publication fees, honoraria for lectures, and travel support to attend educational meetings from Nordberg. Dr. Benítez has no financial interests to declare. Dr. Díaz has no financial interests to declare.

## Supporting information


**Figure S1:** Schematic representation of the subcutaneous injection technique for Poly‐L‐lactic acid (PLLA‐LASYNPRO). Injections were performed using a 25G blunt‐tip cannula with a retrograding (fan‐shaped) technique. The product was reconstituted with 5 mL of sterile saline per vial. A suggested volume of 1.5 cc per side was administered (0.1 cc per injection point). Post‐injection care included applying planar pressure to minimize cord and nodule formation, followed by a 2‐min circular massage to enhance even distribution.

**Figure S2:** Volumetric assessment at 6 months post‐treatment, evaluated using the Vectra H2 system. An increase in volume and projection is observed in the nasolabial fold region (NLF points 1–2–3), indicated by light blue areas. Yellow areas represent a reduction in volume, likely due to traction effects from the treated zones adjacent to NLF 1–2–3.

**Figure S3:** Vectra H2 vector analysis at 6‐month follow‐up. A bilateral soft tissue displacement of approximately 2 mm is observed in the region of the nasolabial folds, indicating sustained traction effects.

**Figure S4:** Volumetric assessment at 6 months post‐treatment, evaluated using the Vectra H2 system. An increase in volume and projection is observed in the nasolabial fold region (NLF points 1–2–3), indicated by light blue areas. Yellow areas represent a reduction in volume, likely due to traction effects from the treated zones adjacent to NLF 1–2–3. The increase in volume/projection of the treated areas has been quantified in cc.

**Table S1:** Summary of efficacy and safety outcomes for the three subjects who discontinued the study at the month 3 visit following completion of all three treatment sessions.

## Data Availability

The data that support the findings of this study are available on request from the corresponding author. The data are not publicly available due to privacy or ethical restrictions.
