# Supplementary figures and images for: Clinical Outcomes of an Innovative Poly‐L‐Lactic Acid (LASYNPRO) in Facial Rejuvenation: Prospective, Multicenter Spanish Study
Source: J Cosmet Dermatol. 2026 Feb 19;25(2):e70753. doi: 10.1111/jocd.70753 (PMC12921352; doi:10.1111/jocd.70753)

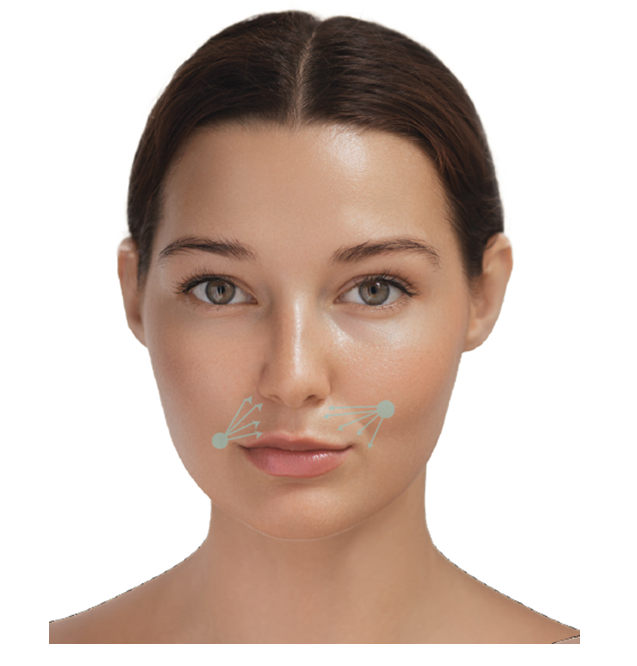

Supplement: Supplementary file 1 — Figure S1: Schematic representation of the subcutaneous injection technique for Poly‐L‐lactic acid (PLLA‐LASYNPRO). Injections were performed using a 25G blunt‐tip cannula with a retrograding (fan‐shaped) technique. The product was reconstituted with 5 mL of sterile saline per vial. A suggested volume of 1.5 cc per side was administered (0.1 cc per injection point). Post‐injection care included applying planar pressure to minimize cord and nodule formation, followed by a 2‐min circular massage to enhance even distribution. [file JOCD-25-e70753-s005.tif]

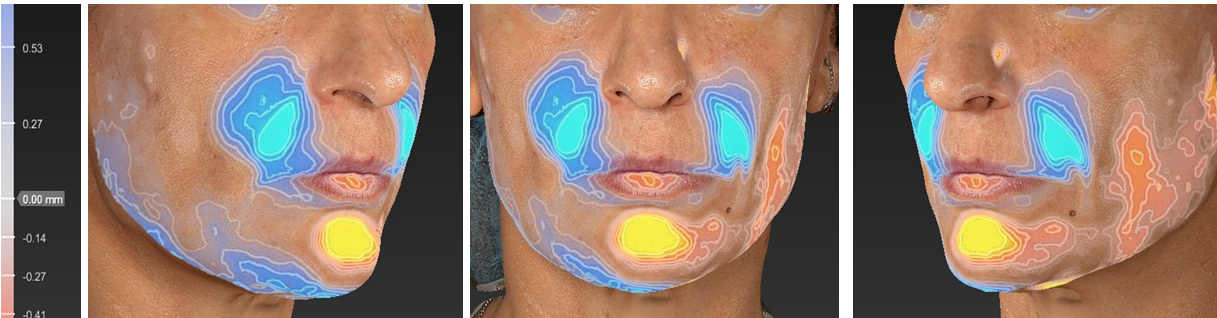

Supplement: Supplementary file 2 — Figure S2: Volumetric assessment at 6 months post‐treatment, evaluated using the Vectra H2 system. An increase in volume and projection is observed in the nasolabial fold region (NLF points 1–2–3), indicated by light blue areas. Yellow areas represent a reduction in volume, likely due to traction effects from the treated zones adjacent to NLF 1–2–3. [file JOCD-25-e70753-s001.tif]

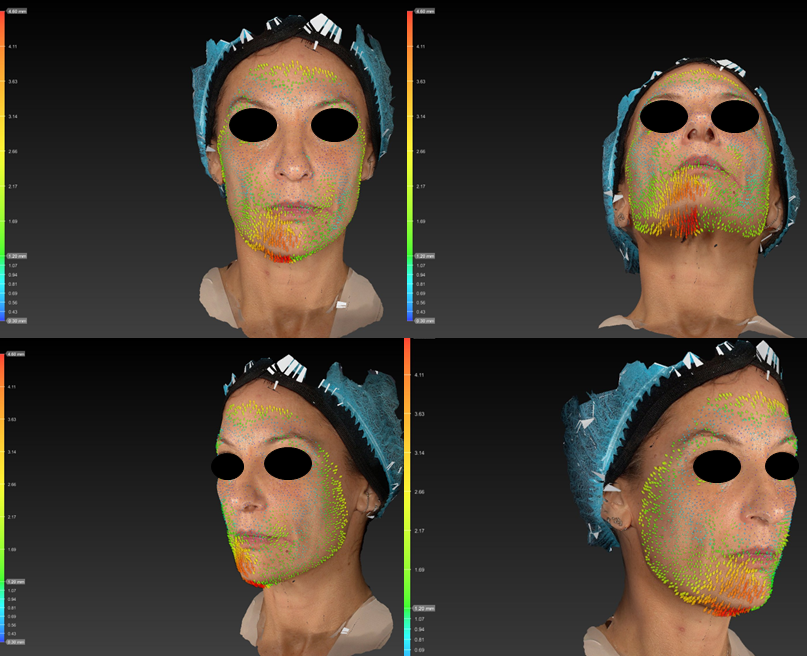

Supplement: Supplementary file 3 — Figure S3: Vectra H2 vector analysis at 6‐month follow‐up. A bilateral soft tissue displacement of approximately 2 mm is observed in the region of the nasolabial folds, indicating sustained traction effects. [file JOCD-25-e70753-s004.tif]

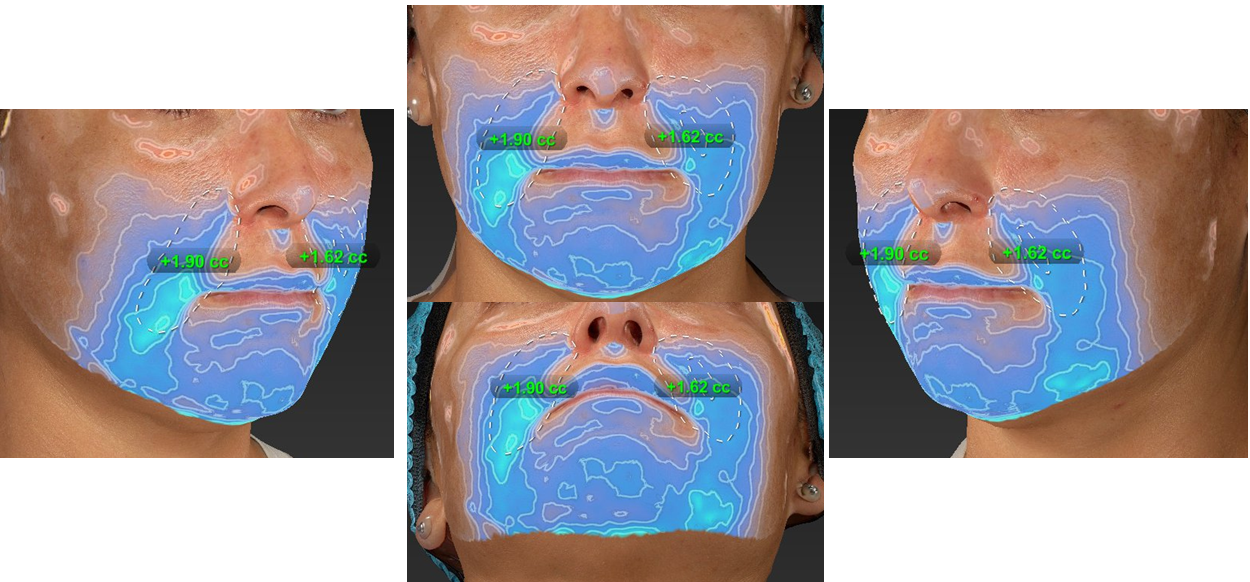

Supplement: Supplementary file 4 — Figure S4: Volumetric assessment at 6 months post‐treatment, evaluated using the Vectra H2 system. An increase in volume and projection is observed in the nasolabial fold region (NLF points 1–2–3), indicated by light blue areas. Yellow areas represent a reduction in volume, likely due to traction effects from the treated zones adjacent to NLF 1–2–3. The increase in volume/projection of the treated areas has been quantified in cc. [file JOCD-25-e70753-s002.tif]
