# Supplementary material for: Clinical Outcomes of an Innovative Poly‐L‐Lactic Acid (LASYNPRO) in Facial Rejuvenation: Prospective, Multicenter Spanish Study
Source: J Cosmet Dermatol. 2026 Feb 19;25(2):e70753. doi: 10.1111/jocd.70753 (PMC12921352; doi:10.1111/jocd.70753)
Supplement: Supplementary file 5 — Table S1: Summary of efficacy and safety outcomes for the three subjects who discontinued the study at the month 3 visit following completion of all three treatment sessions. [file JOCD-25-e70753-s003.docx]

**Clinical Outcomes of an Innovative Poly-L-Lactic Acid (LASYNPRO ^TM^) in Facial Rejuvenation: Prospective, Multicenter Spanish Study.**

**Supplementary Material**

Table S1. Summary of efficacy and safety outcomes for the three subjects who discontinued the study at the Month 3 visit following completion of all three treatment sessions.

| Patients |  |  | Baseline | | | | | Month-3 visit | | | | | | |
| --- | --- | --- | --- | --- | --- | --- | --- | --- | --- | --- | --- | --- | --- | --- |
|  | Center | Age, years | WSRS | | MFVDS | | P1CP | WSRS | | MFVDS | | GAIS | P1CP | AEs |
|  |  |  | Left | Right | Left | Right |  | Left | Right | Left | Right |  |  |  |
| Case 30 | 3 | 45 | 2.5 | 2.5 | 2.5 | 2.5 | 108.0 | 2 | 2 | 2 | 2 | 3^a^ | 125 | No* |
| Case 31 | 3 | 39 | 3 | 3 | 3 | 3 | 152.0 | 2.5 | 2.5 | 2.5 | 2.5 | 3^a^ | 205 | No* |
| Case 32** | 3 | 38 | 3 | 3 | 3 | 3 | 115 | 2.5 | 2.5 | 2.5 | 2.5 | 3^a^ | 117 | No* |
| Case 36 | 3 | 56 | 3 | 3 | 3 | 3 | 189.0 | 2.5 | 2.5 | 2.5 | 2.5 | 2^b^ | 233 | No* |

In the four cases injections were performed using a 25G blunt-tip cannula with a retrograding (fan-shaped) technique. The product was reconstituted with 5 mL of sterile saline per vial. A volume of 1.5 cc per side was injected.

*Mild localized inflammation and erythema were observed immediately post-injection, which resolved spontaneously without the need for medical intervention.

**Last follow-up visit was at month-4.

^a^ Moderate improvement, yet not fully satisfactory.

^b^ No perceptible difference compared to baseline.

WSRS: Wrinkle Severity Rating Scale; MFVDS: Midface Volume Deficit Scale; GAIS: Global Aesthetic Improvement Scale.

**Figure Legends**

Figure S1. Schematic representation of the subcutaneous injection technique for Poly-L-lactic acid (PLLA-LASYNPRO™). Injections were performed using a 25G blunt-tip cannula with a retrograding (fan-shaped) technique. The product was reconstituted with 5 mL of sterile saline per vial. A suggested volume of 1.5 cc per side was administered (0.1 cc per injection point). Post-injection care included applying planar pressure to minimize cord and nodule formation, followed by a 2-minute circular massage to enhance even distribution.

Figure S2. Volumetric assessment at 6 months post-treatment, evaluated using the Vectra H2 system. An increase in volume and projection is observed in the nasolabial fold region (NLF points 1–2–3), indicated by light blue areas. Yellow areas represent a reduction in volume, likely due to traction effects from the treated zones adjacent to NLF 1–2–3.

Figure S3. Vectra H2 Vector Analysis at 6-Month Follow-Up.
A bilateral soft tissue displacement of approximately 2 mm is observed in the region of the nasolabial folds, indicating sustained traction effects.

Figure S4. Volumetric assessment at 6 months post-treatment, evaluated using the Vectra H2 system. An increase in volume and projection is observed in the nasolabial fold region (NLF points 1–2–3), indicated by light blue areas. Yellow areas represent a reduction in volume, likely due to traction effects from the treated zones adjacent to NLF 1–2–3.

The increase in volume/projection of the treated areas has been quantified in cc.
